# Kimura disease with Allergic Bronchopulmonary Aspergillosis: a case report

**DOI:** 10.1186/s13223-022-00683-1

**Published:** 2022-06-27

**Authors:** Ruiyun Fan, Guopeng Xu, Ying Chen, Jinghuan Lv, Zhongwei Zhang

**Affiliations:** 1 Department of Pulmonary and Critical Care, Gusu School, The Affiliated Suzhou Hospital of Nanjing Medical University, Suzhou Municipal Hospital, Nanjing Medical University, Suzhou, 215002 Jiangsu Province China; 2 Department of Pathology, Gusu School, The Affiliated Suzhou Hospital of Nanjing Medical University, Suzhou Municipal Hospital, Nanjing Medical University, Suzhou, 215002 Jiangsu Province China

**Keywords:** Kimura disease, Inguinal lymph nodes, Eosinophilia, Immunoglobulin E, Allergic Bronchopulmonary Aspergillosis

## Abstract

**Background:**

Kimura disease (KD) is a rare chronic idiopathic condition of unknown etiology that is prevalent in Asian males. It often causes subcutaneous lumps and enlarged lymph nodes, especially in head and neck region. But KD is also a systemic disease that can involve multiple organs, such as the kidneys and skin.

**Case presentation:**

We report a 62-year-old Chinese man who presented with paroxysmal cough, enlarged inguinal lymph nodes, recurrent skin itching, and elevated IgE antibodies specific to *A. fumigatus*. After a comprehensive review, the final diagnosis for this patient was KD with Allergic Bronchopulmonary Aspergillosis (ABPA).

**Conclusions:**

The age of onset and the location of the lump involved were not characteristic for the illness. This case report described the patient’s diagnosis and treatment process. This case report serves to arouse the attention of multidisciplinary team to explore the potential relationship between KD and ABPA. It will contribute to preventing the misdiagnosis and missed diagnosis of KD.

## Background

Kimura’s disease is a rare idiopathic, chronic inflammatory but benign disorder which usually presents as painless subcutaneous masses in the head-neck region. Sometimes it also involves extracutaneous sites, such as salivary glands, lymph nodes, skin, and kidneys. It is endemic to Asia and tends to affect young men aged 20–40 years, with some sporadic cases in non-Asians [1]. Two characteristic laboratory examinations are peripheral eosinophilia and elevated serum immunoglobulin E (IgE) [2]. The etiology and pathogenesis of KD are still unclear so far. The diagnosis of KD mainly depends on pathological examination. Treatment should aim to relieve symptoms and preserve aesthetics while preventing recurrences and long-term sequela [3, 4]. In this study, we report a case of KD with ABPA in a Chinese man with special clinical manifestations.

## Case presentation

**Fig. 1:**
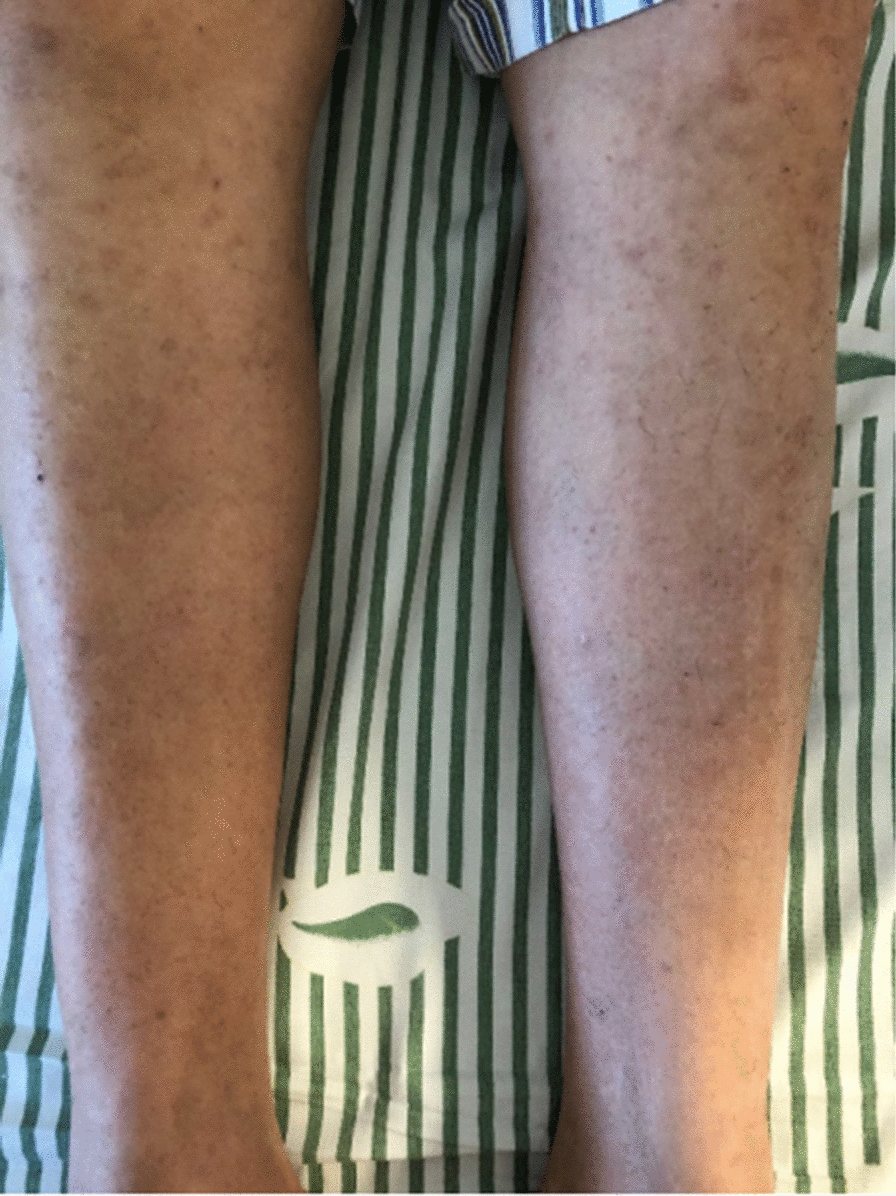
Pigmentation left after red rashes fading on both lower limbs

**Fig. 2:**
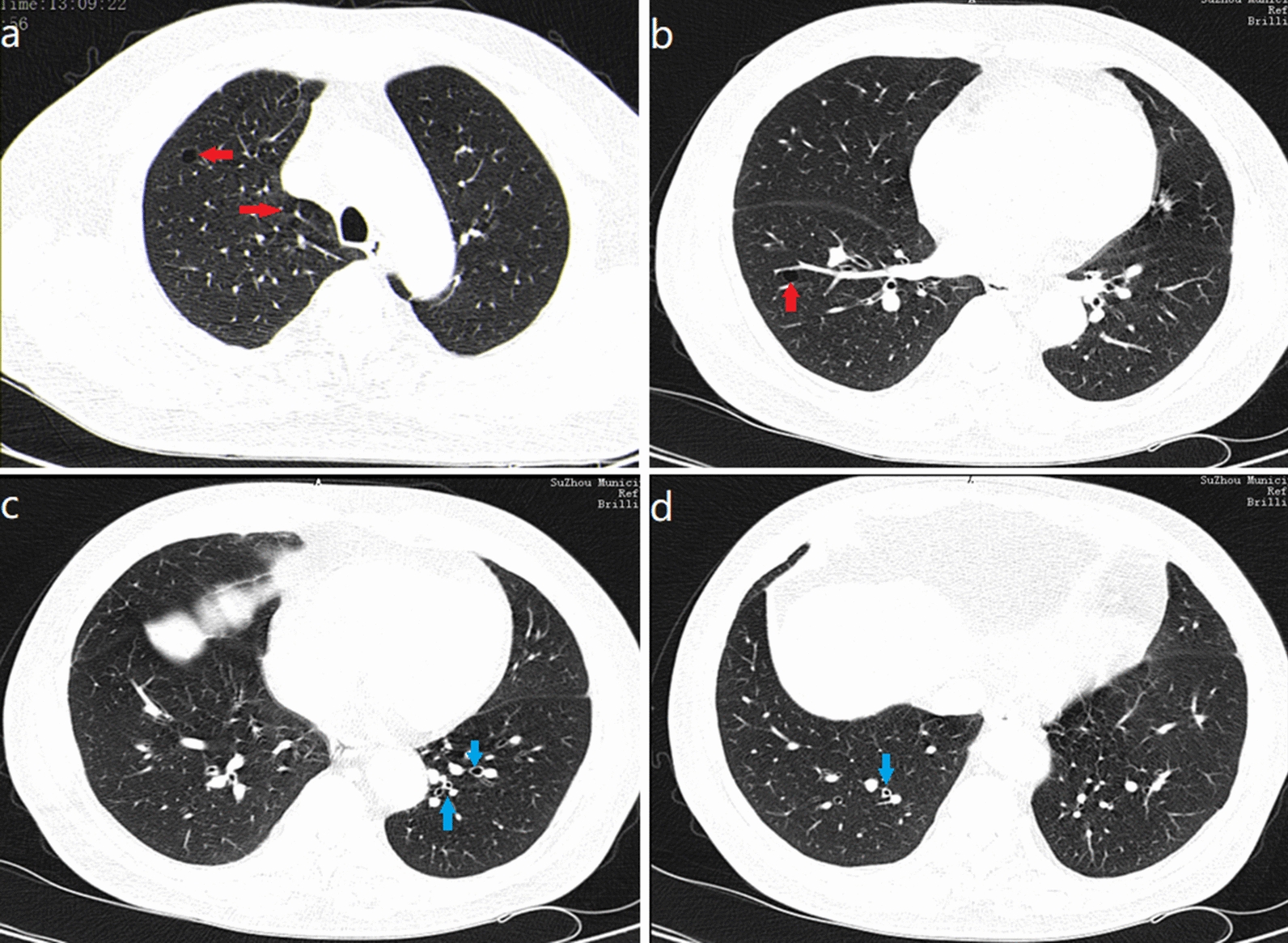
Chest computed tomography (CT) examination: **a**, **b** Increased texture and a little emphysema in both lungs; multiple pneumatoceles (red arrows). **c**, **d** Thickening bronchial walls and mild bronchiectasis in the lower lobe of lungs (blue arrows)

**Fig. 3:**
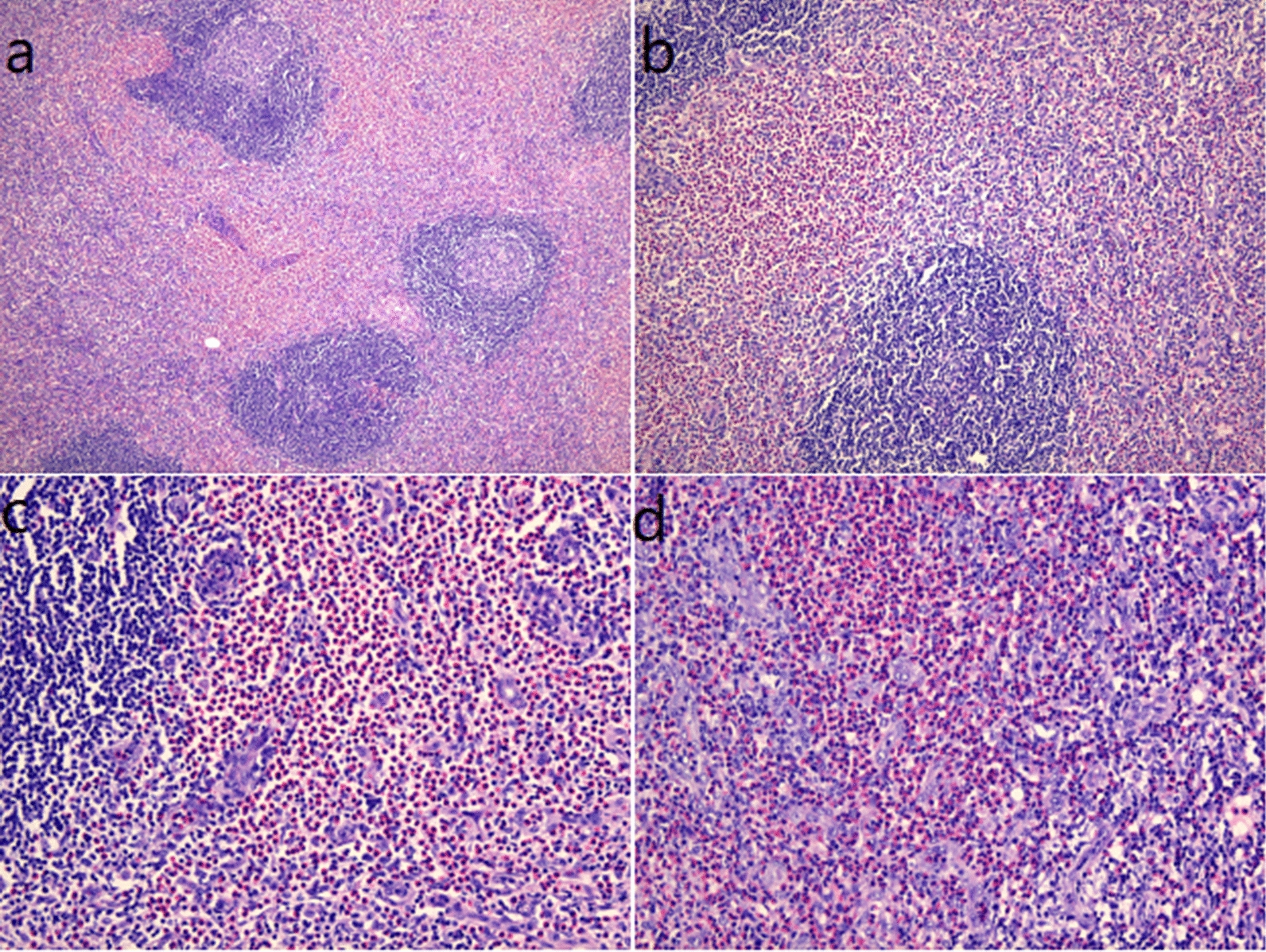
Hematoxylin-and-eosin-stained sections of the right inguinal lymph node biopsy: **a**, **b** Complete lymph node structure, lymph node follicular hyperplasia, and germinal center enlargement. **c**, **d** A large number of eosinophil infiltration and eosinophil abscess in the follicular and interfollicular area

A 62-year-old Chinese man, with a history of coughing and wheezing after being exposed to irritating gases and dust for 50 years, had not received a formal diagnosis and treatment. Four years ago, he presented to the Department of Dermatology because of recurrent itchy skin rashes on the trunk and limbs without obvious triggers. Laboratory tests showed that blood eosinophil count and serum IgE were elevated, then the “eosinophilic dermatose” was diagnosed. Dermatologists recommended long-term oral prednisolone and cetirizine treatment options. After that, skin pruritus and rashes gradually subsided but still occurred repeatedly. Eight months ago, the patient developed a severe cough and wheezing, and found the right inguinal lymph nodes were swollen without redness, pain, and tenderness. He went to the respiratory department of our hospital. Physical examination revealed bilateral wheezes; scattered rashes and marked pigmentation could be seen on the skin of the whole body, mainly on both lower limbs (Fig. 1); several hard, painless masses about the size of quail eggs could be palpated in the right groin area. The patient denied a history of smoking and any family history of malignancy. Hematology revealed total white blood cell (WBC) count of 15.60 × 10E9/l (normal: 3.5–9.5 × 10E9/l), a higher eosinophils count of 5.9 × 10E9/l (normal: 0.02–0.52 × 10E9/l), and the percentage of eosinophils of 37.8% (normal: 0.4–8%). His tumor markers, anti-neutrophil cellular antibody (ANCA), anti-nuclear antibody, and blood GM test were negative, but the fungal G test was positive. Total serum IgE level was 9400 IU/ml (normal < 165 IU/ml) while IgG, IgM, IgA, C3, and C4 were within the normal range. The serum creatinine concentration was 65.0 μmol/L (normal:57–111 μmol/L) and the blood urea nitrogen concentration was 3.66 mmol/L(normal:3.6–9.5 mmol/L); transaminases demonstrated an alanine transaminase of 19 U/L (normal, 9–50 U/L) and an aspartate transaminase level of 16 U/L (normal, 15–40 U/L). Blood tests for thyroid function revealed that the free thyroxine level was 11.65 pmol/L (reference value 11.50–22.70 pmol/L), the free triiodothyronine level was 4.55 pmol/L (reference value 3.50–6.50 pmol/L), the thyroid-stimulating hormone level was 1.1026  μIU/mL (reference value 0.550–4.780  μIU/mL). Coagulation function investigation showed D-dimer was 3.93 mg/L (normal < 0.55 mg/L). Pulmonary function examination revealed that FEV_1_ (forced expiratory volume in one second) was 1.64 L, accounting for 59% of the predicted value, FEV_1_/FVC (forced vital capacity) was 85%, and the increase in FEV_1_ after inhalation of Salbutamol sulphate aerosol was 15% (> 12% pre-bronchodilator) and 250 mL (> 200 mL) from baseline. Chest computed tomography (CT) examination showed increased texture and a little emphysema in both lungs, and multiple pneumatoceles in the right lung. We also found several bronchial walls were thickened and slightly dilated in the lower lobe of lungs (Fig. 2). Vascular ultrasound (US) examination of the vessels showed femoral atherosclerotic plaque formation in both lower extremities. Inguinal ultrasound examination revealed several enlarged lymph nodes in the right groin. We considered that right inguinal lymphoma couldn’t be ruled out, so the patient was recommended to undergo a lymph node biopsy. Pathological results showed that complete lymph node structure, lymph node follicular hyperplasia, germinal center enlargement, a large number of eosinophil infiltration and eosinophil abscess in the follicular and interfollicular area, which was consistent with the diagnosis of KD (Fig. 3). Immunohistochemical staining showed that LCA, Vimentin was positive, CD2, CD79a, CD3, CD5, CD43, CD21, CD23, CD68, Ki-67 was partially positive, S-100 was sporadically positive, and CD1, CD123, Lingren was negative.

Because of his positive fungus G test result and symptoms of bronchial asthma, we suspected that ABPA would be diagnosed. Further inspection revealed that IgE specific to *Aspergillus. fumigatus* was 1.36KUA/L (normal < 0.1KUA/L). It should be noted that the patient had symptoms of bronchial asthma and met two obligatory criteria of elevated IgE levels against Aspergillus fumigatus (> 0.35 KUA/L) and elevated total IgE levels (> 1000 IU/mL). Moreover, he met two secondary criteria of blood eosinophils count > 0.5 × 10E9/L and mild bronchiectasis on imaging, so he was diagnosed as ABPA [5].

**Fig. 4:**
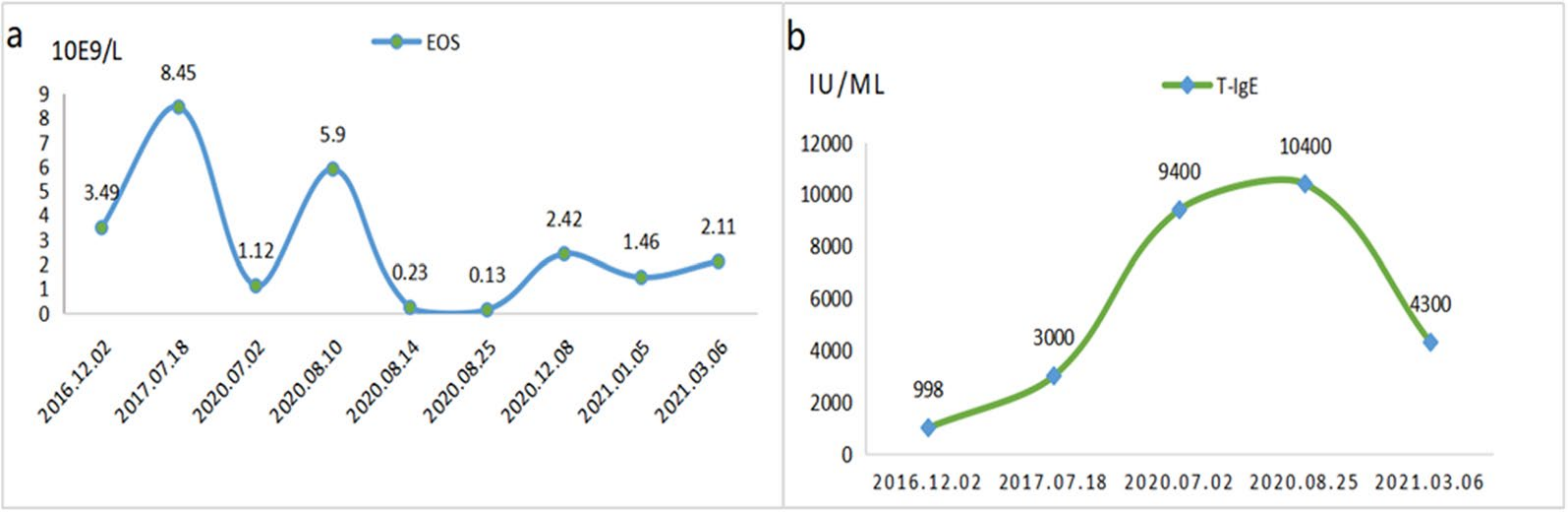
**a** The change curve of peripheral eosinophils. **b** The change curve of and serum total IgE Level

Considering that the enlarged inguinal lymph nodes were small and had no obvious symptoms of discomfort, the patient refused surgical resection and finally accepted conservative treatment. According to his condition, we recommended omalizumab as the first choice. He refused and chose oral prednisone (40 mg/day) combined with itraconazole (200 mg twice daily). The patient was discharged after 3 weeks, when the dose of prednisone had been reduced to 20 mg per day. Before leaving the hospital, the hematologic parameters were checked again. The WBC count was 18.60 × 10E9/l with 0.7 percent eosinophils (absolute value: 0.13 × 10E9/l). The total serum IgE level was increased (9400 IU/ml). He had no obvious cough and asthma. The patient was followed up regularly to adjust the dose of the drugs. From January 2021, he started taking prednisone (10 mg/day) combined with itraconazole (100 mg /day) for maintenance treatment. We plotted the changes of his peripheral blood eosinophil count and serum total IgE into a curve (Fig. 4). The fluctuations were not directly related to the adjustment of the drug dose. His previously swollen inguinal lymph nodes were currently not palpable and there were no signs of disease progress-ion. The patient was satisfied with the current curative effect and had a high compliance. No adverse drug reactions and relapses have occurred up to now.

## Discussion and conclusions

KD was first described as "eosinophilic hyperplastic lymphogranuloma” by Kimm and Szeto (China) in 1937, but it was first reported definitively by Kimura (Japan) in 1948 and has been widely known as Kimura disease since then [6]. It is endemic to Asia and tends to affect young men aged 20–40 years. But it has no strict restrictions on ethnicity, age, and gender. The previous reports have showed that sporadic cases were also found in Spain, Saudi Arabia, South America, and other non-Asians. The youngest patient was a 15-month old African American boy [7]. Although it is recognized that two typical laboratory examinations are elevated peripheral eosinophil and serum IgE, Vivek et al. reported a case of KD without peripheral eosinophilia [4]. KD is characterized by subcutaneous masses in the head and neck region, sometimes with local lymph node enlargement and salivary gland involvement. Rare cases can involve the orbit and ocular appendages, epiglottis, armpit, long bone, breast, groin, genitals, mediastinum. In this case, the patient’s age was not consistent with the classic age of onset reported in previous literature. Our patient presented rare multiple enlarged inguinal lymph nodes. KD is a systemic disease that can usually involve kidneys and skin, which are usually manifested as nephrotic syndrome, skin pruritus, or rashes, respectively. Our patient had normal liver and kidney function, but there were obvious itching and rashes on the trunk and extremities. It had been reported that eosinophils contain various amounts of tissue factor (TF) and some researchers had concluded that relatively high TF in patients with hypereosinophilia might lead to an increased thrombotic risk [8]. A patient with KD was reported to be complicated with acute limb ischemia and coronary artery disease [9]. The D-dimer in the patient we reported was at a high level. Vascular US examination of both lower extremities showed bilateral femoral atherosclerotic plaques formation, suggesting he had a high risk of thrombosis. Therefore, with the consent of the patient, we prescribed a 0.4 ml heparin sodium injection for prophylactic anticoagulation. The coagulation function result revealed D-dimer was 1.5 mg/L before he was discharged from the hospital.

**Fig. 5:**
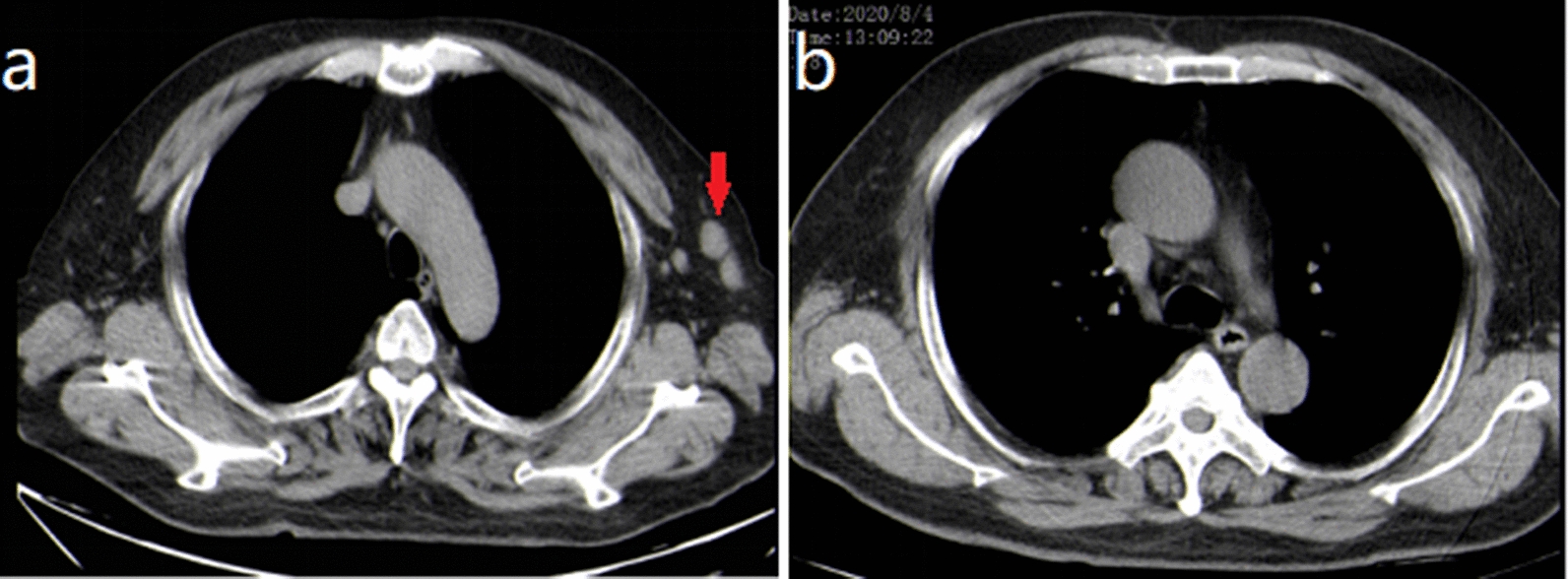
Chest CT scan: **a** Several enlarged lymph nodes could be seen in the left armpit in December 2018 (red arrow). **b** No enlarged lymph nodes found in the left armpit in August 2020

KD is a benign disease of unknown etiology and pathogenesis. However, the increased peripheral eosinophil and serum IgE have oriented that KD might be associated with autoimmunity, insect bites or infections, and allergies caused by parasites [10, 11]. Otah N et al. have revealed that Th2 and Tc1 cells instead of Th1 and Tc2 cells contribute to the pathogenesis of KD [12]. Katagiri K et al. have found that the expression levels of IL-4, IL-5, IL-13, and IFN-γ mRNAs in peripheral blood mononuclear cells in a patient with KD are significantly higher than after surgery or radiotherapy, which supports Th2 cytokines play a role in the development of KD [13]. In addition, Agarwal R et al. have reviewed that the immune response in ABPA is a Th2 CD4^+^ T cell response, accompanied by increased secretion of cytokines such as IL-4, IL-5, and IL-13. The Th2 immune response leads to the synthesis eosinophils and IgE (including total and A. fumigatus specific) [5]. So we suspected that there was a certain correlation between the pathogenesis of these two diseases rather than an incidental. To our knowledge, this is the first case of KD combined with ABPA. Based on the order of onset of the two diseases, we have reasonable speculation that ABPA may induce the occurrence of KD, but the speculation needs more clinical evidence to confirm. When reviewing the patient's history, we unexpectedly found the patient’s chest CT examination showed several enlarged lymph nodes in the left axilla as early December 2018 (Fig. 5a). He didn’t have any relative discomfort at that time, it subsided without symptomatic treatment in August 2020 (Fig. 5b). This time the right inguinal lymph nodes enlargement occurred, which manifested KD was self-limited and recurrent with an indolent course and good prognosis [14].

The definite diagnosis of KD depends on histopathologic examination. Our initial diagnosis suspicion was lymphoma, but the histopathologic results helped us get an accurate diagnosis. The imaging findings, including CT, magnetic resonance imaging (MRI), and US are non-specific, but they are helpful to deter-mine the lesion morphology, anatomical distribution. Yang et al. indicated that the ill-defined, infiltrative lesions on imaging were associated with a higher recurrence rate [15]. Differential diagnosis of KD includes angiolymphoid hyperplasia with eosinophilia (ALHE), cutaneous IgG4-related disease (IgG4-RD), Hodgkin's lymphoma, Langerhans cell histiocytosis, florid follicular hyperplasia, Castleman's disease, dermatopathic lymphadenopathy [16]. The main differential diagnosis is ALHE which shares similar histological features with KD, however, patients with ALHE typically have normal eosinophils and IgE level [17].

There is no standard treatment protocol for KD to date. Treatment includes surgical resection, regional or systemic steroids, antihistamines, immunosuppressant including cyclosporine (CsA) or anti-IgE antibody (omalizumab), or radiotherapy [18, 19]. The individualized treatment plan should be selected according to the clinical manifestation of patients. For patients with KD combined with renal involvement, steroids may be the best treatment [20]. Surgical resection is preferred for primary lesions without multiple system involvement [21].

Kimura disease is a chronic disease with a prolonged course and a high recurrence rate. No malignant transformation has been reported till date. KD involves multiple disciplines such as immunology, dermatology, nephrology, and otolaryngology. Its different clinical manifestations have varying degrees of impact on patient’s lives, including pruritus, renal function damage, disfigurement, vascular embolism. Rare cases and atypical manifestations of KD make the early diagnosis difficult. Our unique case may give some clinicians the inspiration to explore the relationship between KD and ABPA. Multidisciplinary team should pay more attention to collect clinical data to study the etiology of KD.

## Data Availability

Data sharing is not applicable to this article, as no datasets were generated or analyzed during this case report.

## Abbreviations

KD: Kimura disease
ABPA: Allergic Bronchopulmonary Aspergillosis
IgE: Immunoglobulin E
WBC: White blood cell
ANCN: Anti-neutrophil cellular antibody
FEV_1_: Forced expiratory volume in one second
FVC: Forced vital capacity
CT: Computed tomography
US: Ultrasound
MRI: Magnetic resonance imaging
TF: Tissue factor
ALHE: Angiolymphoid hyperplasia with eosinophilia
IgG4-RD: IgG4-related disease
CsA: Cyclosporine
